# A commentary on the XIII^th^ International Rotifer Symposium (Shillong, 2012)

**DOI:** 10.1186/2046-9063-9-13

**Published:** 2013-07-01

**Authors:** Robert Lee Wallace, SSS Sarma, S Nandini

**Affiliations:** 1 Department of Biology, Ripon College, Ripon, WI 54971-0248, USA; 2 Laboratorio de Zoología Acuática, Edificio UMF, Universidad Nacional Autónoma de México, Campus Iztacala, Av. de los Barrios, no. 1, Los Reyes, Tlalnepantla, Edo. de Méx. CP 54090, Mexico

**Keywords:** Aging, Aquaculture, Cryptic speciation, Ecology, Ecotoxicology, Genetics, Taxonomy

## Abstract

Rotifers have attracted the attention of biologists for well over 200 years. Interest in these exquisite animals rests in their diverse morphology, short generation time resulting in high growth rates, ability to withstand desiccation, and wide distribution, coupled with evidence of cryptic speciation. Moreover, three modes of reproduction are present in the phylum: obligatory sexuality, cyclical parthenogenesis, and obligatory ameiotic parthenogenesis. Thus, this phylum offers a rich field of study. Recognizing the need to share advances in knowledge, a triennial meeting, the International Rotifer Symposium (IRS), was begun in 1976. The most recent symposium (13^th^ IRS) was held at Shillong (India) from 18–24, November 2012. In this commentary we considered the development of rotifer research as viewed through the lens of more than 35 years of IRS. Initially papers presented at the IRS focused on ecology, morphology, and pure taxonomic problems, with little applied work being reported. However, after more than three decades, the emphasis has swung to a balance of both basic (e.g., aging, ecology, genetics, and taxonomy) and applied (aquaculture and ecotoxicology) research.

## Findings

Unlike marine systems, which possess a rich array of multicellular zooplankton, three groups dominate freshwaters: rotifers, cladocerans, and copepods [1,2]. With >2,000 described species, rotifers are particularly interesting to aquatic ecologists due to their high species diversity, curious modes of reproduction (ameiotic parthenogenesis in bdelloids vs. cyclical parthenogenesis in monogononts), short generation time (≤5 days), and high growth rates (r_max_ ≥1.0). The fact that males are unknown in bdelloids is another curiosity that has won them the label of an “evolutionary scandal” [3]. In addition, the adults and embryos of some bdelloids and the diapausing embryos of monogononts are capable of surviving anhydrobiosis and dispersing in this state.

Appreciating the importance of rotifers in modern biology, Agnes Ruttner-Kolisko offered a suggestion at the 1974 International Society of Limnology (SIL) congress: that an international meeting of workers interested in exchanging ideas on rotifers be organized. Her dream of the first International Rotifer Symposium (IRS) was made real in the early fall of 1976 in Lunz am See (Austria) [4]. Thereafter, the enthusiasm among the rotifer workers fused into a loose society — “the rotifer family”, as H.J. Dumont calls it. To date 13 rotifer symposia have been held in different parts of the world (Table 1).

**Table 1:** **Details of the International Rotifer Symposia** (**1**–**13**)

**Symposium**	**Date and place**	**Host**	**Total participants**	**Countries represented**
**I**	21-26 Sept, 1976; Lunz, Austria	Ruttner-Kolisko A	38	15
**II**	17-21 Sept. 1979; Ghent, Belgium	Dumont HJ	51	16
**III**	30 Aug. - 4 Sept. 1982, Uppsala, Sweden,	Pejler B	70	22
**IV**	18-25 Aug. 1985, Edinburgh, Scotland, UK	May L	68	23
**V**	12-17 Sept. 1988, Gargnano, Italy	Ricci C	83	20
**VI**	3-8 June 1991, Banyoles, Spain	Miracle MR	107	25
**VII**	6-11 June 1994, Mikolajki, Poland	Ejsmont-Karabin J	93	26
**VIII**	22-27 June 1997, Collegeville, Minn. USA	Wurdak E	97	22
**IX**	16-23 Jan 2000, Khon Kaen, Thailand	Sanoamuang L-o	117	26
**X**	7-13 June 2003, Illmitz, Austria	Herzig A	113	28
**XI**	11-18 March 2006, Mexico City, Mexico	Sarma SSS	125	20
**XII**	16-21 August 2009, Berlin, Germany	Walz N	136	30
**XIII**	18-24, November 2012, Shillong, India	Sharma BK	65	20

### Highlights of the 13^th^ International Rotifer Symposium

**Figure 1:**
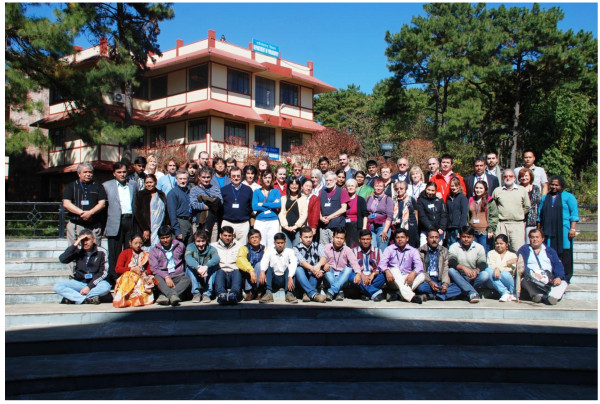
Participants of the XIII International Rotifer Symposium (Shillong, India, 2012).

The XIII^th^ IRS was held on the campus of the North-Eastern Hill University in the State of Meghalaya, India during the week of 18–24 November, 2012 (Figure 1). Although the number of rotifer workers who participated in this meeting was lower than in most of the previous ones, the enthusiasm among the participants was just as high. This interest was reflected in the themes chosen for the meeting. There were seven technical sessions, each with two chairpersons. In addition, there were seven invited talks, two poster sessions, two workshops, and a full-day field trip, all within a 5-day conference; this made the entire week densely packed. The technical sessions began with presentations on morphological and taxonomy of rotifers. As molecular tools have considerably advanced since the last meeting, most works on taxonomy now use these tools to investigate the phylogenetic relationships among taxa and the concept of cryptic speciation within the phylum.

The invited talks took into account the state of the art of several of the main areas of contemporary rotifer research. The first presentation was on the rotifers of temporary waters, habitats where life is adapted to the duel existence of a wet–dry cycle. This overlooked field is becoming of more interest to aquatic ecologists due to the prediction that climate change will increase desertification, which will result in a loss of ecosystem services [5]. The presenters showed that these waters comprise a remarkably high diversity of rotifers. The second invited lecture complemented the first in that it presented an update on the status of an international effort began at the Berlin symposium (XII^th^ IRS) to develop a comprehensive list of available names in Rotifera according to the rules of the International Code of Zoological Nomenclature [6]. Other invited talks included three that dealt with basic ecological aspects (rotifer body size, aging process, and life histories) and one dealing with applied rotifer research in its application to aquaculture. The presentations during the technical sessions were very diverse, embracing various themes, including morphology, molecular biology, population and community structure, the use of rotifers as ecological indicator species, and their use in bioassays studies.

As in several of the previous symposia, the XIII^th^ meetings had two workshops: one was a basic primer on sessile rotifers and the other was on using rotifers in ecotoxicology research. The workshops began with all participants being briefed by E.J. Walsh, H. Segers, and R.L. Wallace on questions regarding the sessile species; as part of these presentations Segers showed some spectacular videos that were done by P. Mesuwan, a Thai student. For many of the participants this was the first time that they had an opportunity to view sessile rotifers, which are often unidentifiable in preserved state. The participants then separated and the two workshops continued in parallel. In the ecotoxicology workshop, S.S.S. Sarma provided an overview of the use of rotifers in ecotoxicological bioassays, including a review of the species currently in use for these tests. S. Nandini reported on the application of bioassay to cyanotoxicity studies and the control of cyanobacterial blooms. M.R. Miracle provided information on modifications to current test procedures.

## Discussion

The participants of the IRS are not insular in their studies; that is, elucidating rotifer biology is not the sole purpose of these meetings. Indeed the research presented at these meetings explores the ecological and evolutionary processes that structure freshwater communities and drive the evolution of rotifers at micro- and macro-scales. However, the topics covered have not been evenly represented through the years. Nevertheless, several prominent themes have emerged or continue to be developed that are of interest to aquatic ecologists in general. These included aquaculture, bdelloid biology (anhydrobiosis and genetics), biogeography and dispersal, community assembly, ecotoxicology, molecular biology, population ecology (especially with regard to abiotic drivers), and taxonomy based on both morphology and molecular sequences.

Ecotoxicology is one of the two applied aspects of rotifer research that has been gaining momentum. Snell and Janssen [7] presented the first comprehensive review of rotifers in ecotoxicology at the VII^th^ IRS at Spain, and at the XI^th^ IRS at Mexico City Snell and Joaquim-Justo [8] provided an updated version on this topic. At the Shillong meeting many recent advances on the use of rotifers as bioassay organisms were presented including, their use in evaluating cyanotoxin toxicity, as well as the use of rotifers as indicator species. In the discussions that followed the suggestion was made that different rotifer species including *Lecane* and *Proales*, be employed as possible new candidates for bioassays. Attention also was paid to the lack of choice of taxa to be used in marine ecotoxicity testing. At present, considerable data on this aspect is available from the *Brachionus plicatilis* species complex, which is not necessarily the most sensitive among the marine rotifers. Many other genera of marine rotifers such as *Encentrum* and *Synchaeta* are probably highly sensitive to toxicants, but unfortunately quantitative data on these species is lacking.

A comparison of the progress reported over the course of 13 IRS meetings reveals several interesting facts. For example, during the I^st^ IRS the emphasis was on ecology of rotifers, with more than 80% of the works dealing with this aspect. On the other hand, applied work, such as aquaculture and ecotoxicology, was hardly represented. Now after more than three decades of meetings, the emphasis appears to be balanced. In fact, during the last two symposia, there were separate presentations on aquaculture and ecotoxicology. Of course, taxonomic questions continue to be presented at the meetings, but an important shift has been taking place, especially in the use of molecular tools to discover cryptic species [9-12]. Nonetheless, it is unlikely that conventional morphological taxonomy will be completely replaced by molecular taxonomy, at least for the foreseeable future. However, there is a practical aspect to classical morphological studies in terms of career development. Unfortunately, the editors of journals with high impact factors are not favourably disposed to publish manuscripts based on purely taxonomic questions; one reason for that is the number of citations per article on these topics is low. Yet, science is on the horns of a dilemma here – without reliable classical α-taxonomy, results from any line of research are suspect [6,13,14].

The importance of molecular tools in establishing phylogenetic relationships within the Rotifera is now well established [15,16]. Such tools are becoming increasing popular to investigate the phylogenetic relationships among animal phyla. In this context, one of us (RLW) suggested that an effort be made to invite researchers who work on phylum Acanthocephala to future meetings. Given that molecular evidence supports the hypothesis that Rotifera and Acanthocephala are closely related phylogenetically, it seems likely that participation of acanthocephalan researchers in an IRS will enhance our knowledge on the evolution of lower invertebrates.

While research on bdelloids was not presented at the first IRS, the importance of this group has been recognized for a variety of reasons. Chief among these are our understanding that (1) males have been absent for > 40 million years, (2) several species are capable withstanding extremely high doses of radiation with subsequent repair of double stranded breaks in their DNA, (3) the adults of some species are capable of undergoing anhydrobiosis, and (4) bdelloids incorporate fragments of foreign DNA into their genome. During the last 35+ years, bdelloids have been the subject of intense research and many new insights into their biology and genetics have been presented during the IRS.

As in all modern scientific meetings, an important part of the IRS lies in its poster sessions. Unlike oral presentations, where a speaker’s time is limited, authors of the poster sessions remain as long as a viewer needs them. Thus, many young researchers have been using the poster sessions because they are able to interact more closely with other participants to gain insights on how to improve their research. Usually the poster sessions at the IRS are organized at pleasant sites (e.g., on the banks of a lake, at the XI^th^ IRS at Lake Xochimilco, Mexico City). At the Shillong meetings the posters were displayed in the same hall as all the talks. This permitted the participants to have unlimited access to view the posters during session breaks.

**Figure 2:**
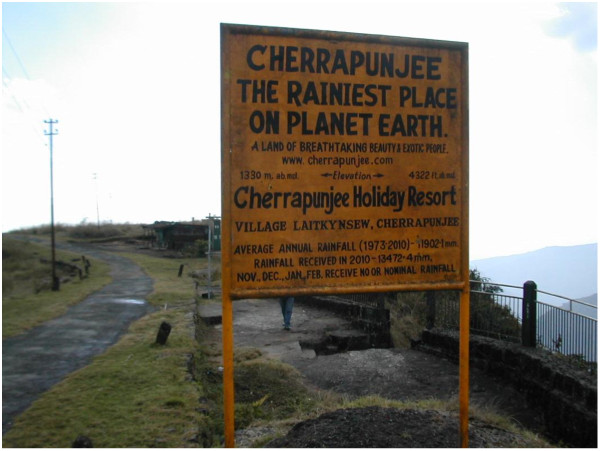
Cherrapunjee, Meghalaya, India.

**Figure 3:**
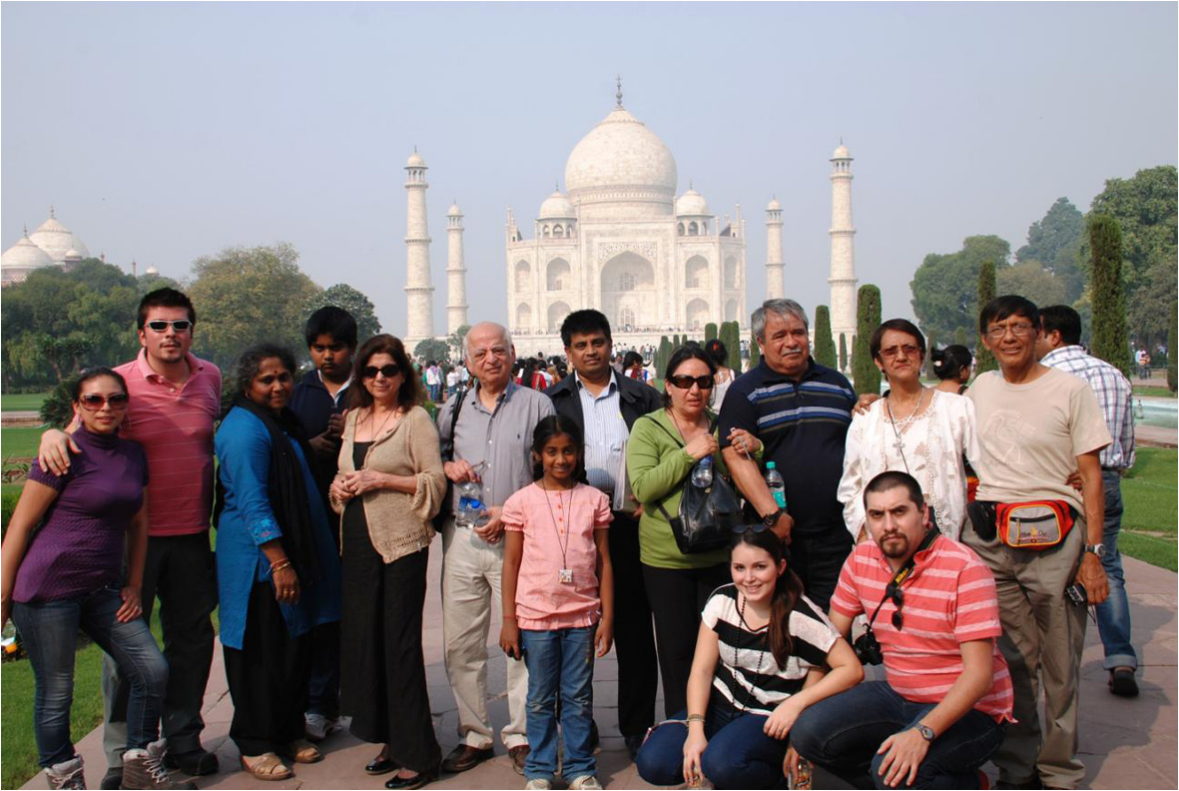
**Rotifer Symposium tourists.** Mexican participants with Dr Ramesh D. Gulati (sixth from left to right) and his wife Toshi Gulati (fifth from left to right) at the historical Taj Mahal visit before the Symposium.

Historically the IRS offers a time for sightseeing during a mid-conference excursion. There were two remarkable trips during the XIII^th^ IRS. One was a visit to Cherrapunjee, one of the wettest places on the earth, having a recorded mean annual rainfall in excess of 10,000 mm per year (Figure 2). The second visit was to the City museum of Shillong, where the museum director provided the participants with explanations of many of the displays of local and regional culture. India also offered much in the way of general tourist interests, and many participants took advantage of this by arriving earlier or staying later to visit such interesting places as the Kaziranga National Park, which boasts of the largest population of the single-horned rhinoceros, and historical monuments in and around New Delhi (Figure 3).

As in all scientific fields, the rotifer community is undergoing change: older researchers retire, but leave the field in the hands of those that they have already trained. Such replenishment is vital for maintenance of a vibrant scientific community. Nevertheless, during the XIII^th^ IRS, four of the 38 participants of the first IRS were present (E. Wurdak, R.L. Wallace, T.W. Snell and M.R. Miracle), and two of those (Snell and Wallace) have attended all the 13 IRS. The rotifer community at Shillong honoured these workers with a memento depicting “Eminent Rotiferologist”. In addition, as a token of respect, mementos were individually presented to a few other rotifer workers too, including L. May, M.R. Miracle, H. Segers, S.S.S. Sarma, B.K. Sharma, S. Sharma, A. Herzig, A. Hagiwara, E. Wurdak and J. Ejsmont-Karabin.

The IRS also distinguishes itself from many other meetings with a continuous record of publishing the proceedings. With the exception of the 1^st^ IRS, the proceedings have appeared in *Hydrobiologia* (Table 2). However, the proceedings of the XIII^th^ IRS will be edited by B.K. Sharma, H.J. Dumont, and R.L. Wallace and published in the *International Journal of Hydrobiology*.

**Table 2:** Details of the proceedings of the 13 International Rotifer Symposia

**Symposium**	**Guest editors**	**Journal details (vol., year)**	**No**. **of papers published**
**I**	King CE	Arch. Hydrobiol. Beih. 8, 1977	52
**II**	Dumont HJ & Green J	Hydrobiologia, 73, 1980	42
**III**	Pejler B, Starkweather P* & Nogrady Th	Hydrobiologia, 104, 1983	52
**IV**	May L, Wallace RL & Herzig A	Hydrobiologia, 147, 1987	50
**V**	Ricci C, Snell TW & King CE	Hydrobiologia, 186/187, 1989	52
**VI**	Gilbert JJ, Lubzens E & Miracle MR	Hydrobiologia, 255/256, 1993	72
**VII**	Ejsmont-Karabin J & Pontin RM	Hydrobiologia, 313/314, 1995	53
**VIII**	Wurdak E, Wallace RL & Segers H	Hydrobiologia, 387/388, 1998	64
**IX**	Sanoamuang L-o, Segers H, Shiel RJ & Gulati RD	Hydrobiologia, 446/447, 2001	51
**X**	Herzig A, Gulati RD, Jersabek CD & May L	Hydrobiologia, 546, 2005	58
**XI**	Sarma SSS, Gulati RD, Wallace RL, Nandini S, Dumont HJ & Rico-Martínez R	Hydrobiologia, 593, 2007	25
**XII**	Walz N, Adrian R, Gilbert JJ, Monaghan MT, Weithoff G & Zimmermann-Timm H	Hydrobiologia, 662, 2011	26
**XIII**	Sharma BK, Dumont HJ & Wallace RL	Int. Rev. Hydrobiol.	In progress

* – The initial for the second editor was incorrectly cited as “R” on the cover and inside cover page.

## Conclusions

It is evident to us that the rotifer meetings have become an important venue for several reasons, chief among these are the establishment of academic and personal contacts, the identification of emerging areas of research, and the development of collaborative projects. In this respect, the plea put forward by E.J. Walsh and H.S. Segers for resolving the *Brachionus plicatilis* species complex is a welcome venture. Many participants of the XIII^th^ IRS also have supported this idea and a collaborative work is already in progress.

Agnes Ruttner-Kolisko’s farsightedness in suggesting a symposium focusing on rotifers has become a successful reality; the rotifer family is indebted to her for this initiative and for making the first symposium a successful model for all that followed. With this recognition, we suggest the inclusion of two invited lectures in all future IRS: the “Ruttner-Kolisko Invited Lecture” on ecological research and the “Koste Invited Lecture” on morphological–taxonomic research.

## Competing interests

The authors declare that they have no competing interests.

## Authors’ contributions

The authors’ contributions for preparing this manuscript are nearly identical. Their order merely reflects professional seniority. All authors read and approved the final manuscript.
